# Comparison of iTRAQ and SWATH in a clinical study with multiple time points

**DOI:** 10.1186/s12014-018-9201-5

**Published:** 2018-07-30

**Authors:** Antti Jylhä, Janika Nättinen, Ulla Aapola, Alexandra Mikhailova, Matti Nykter, Lei Zhou, Roger Beuerman, Hannu Uusitalo

**Affiliations:** 1 Department of Ophthalmology, SILK, The Centre for Proteomics and Personalized Medicine (PPM), Faculty of Medicine and Life Sciences, University of Tampere, Arvo Ylpön katu 34, ARVO, PL 100, 33014 Tampere, Finland; 2 BioMediTech, Faculty of Medicine and Life Sciences, University of Tampere, Tampere, Finland; 3 Singapore Eye Research Institute, Singapore, Singapore; 4 Duke-NUS SRP NBD, Singapore, Singapore; 5 Department of Ophthalmology, Yong Loo Lin School of Medicine, National University of Singapore, Singapore, Singapore; 6 Ophthalmology and Visual Sciences Academic Clinical Research Program, Duke-NUS Medical School, Singapore, Singapore; 7 Tays Eye Center, Tampere University Hospital, Tampere, Finland

## Abstract

**Background:**

Advances in mass spectrometry have accelerated biomarker discovery in many areas of medicine. The purpose of this study was to compare two mass spectrometry (MS) methods, isobaric tags for relative and absolute quantitation (iTRAQ) and sequential window acquisition of all theoretical fragment ion spectra (SWATH), for analytical efficiency in biomarker discovery when there are multiple methodological constraints such as limited sample size and several time points for each patient to be analyzed.

**Methods:**

A total of 140 tear samples were collected from 28 glaucoma patients at 5 time points in a glaucoma drug switch study. Samples were analyzed with iTRAQ and SWATH methods using NanoLC-MSTOF mass spectrometry.

**Results:**

We discovered that even though iTRAQ is faster than SWATH with respect to analysis time per sample, it loses in sensitivity, reliability and robustness. While SWATH analysis yielded complete data of 456 proteins in all samples, with iTRAQ we were able to quantify 477 proteins in total but on average only 125 proteins were quantified in a sample. 283 proteins were common in the datasets produced by the two methods. Repeatability of the methods was assessed by calculating percent relative standard deviation (% RSD) between replicate MS analyses: SWATH was more repeatable (56% of proteins < 20% RSD), compared to iTRAQ (43% of proteins < 20% RSD). Despite the overall benefits of SWATH, both methods showed less than 1 log fold change difference in the expression of 74% common proteins. In addition, comparison to MS/MS peptide results using 8 isotopically labeled peptide standards, SWATH and iTRAQ showed similar results in terms of accuracy. Moreover, both methods detected similar trends in a longitudinal analysis of protein expression of two known tear biomarkers.

**Conclusions:**

Overall, we conclude that SWATH should be preferred for biomarker discovery studies when analyzing limited volumes of clinical samples collected at multiple time points.

**Trial Registeration:**

The study was approved by the Ethics Committee at Tampere University Hospital and was registered in EU clinical trials register (EudraCT Number: 2010-021039-14).

**Electronic supplementary material:**

The online version of this article (10.1186/s12014-018-9201-5) contains supplementary material, which is available to authorized users.

## Background

Biomarkers can reveal a patient’s risk to certain disease or complication and its severity, and predict the therapeutic response [1]. In recent years mass spectrometry (MS) has become an attractive choice for biomarker discovery, because it can be used to detect and quantify most proteins in a given sample [2, 3] and it can thus detect changes in cellular functions and metabolism in a more comprehensive way than the traditional immunoassays [4]. In ophthalmology, tissue samples are always very limited in amount due to the size of the eye and potential damage to healthy structures. However, the tear fluid is a thin layer of extra-cellular fluid over the surface of the eye [5] that can be collected non-invasively. MS analysis of tear samples has proven its potential in discovering proteomic biomarkers and even small quantities of tear fluids are sufficient if collected and analyzed carefully [6–9]. Tear fluid proteomics is relevant for understanding the outcomes of topically applied drugs, adverse reactions, anterior segment surgery, inflammatory diseases such as dry eye as well as other eye and systemic diseases [8–10].

iTRAQ or isobaric tags for relative and absolute quantitation [11, 12] has often been the method of choice for relative quantitation, but more recently a new technique, sequential windowed acquisition of all theoretical fragment ion mass spectra (SWATH) [13], has begun to challenge that position as an alternative method. SWATH and iTRAQ provide relative quantitation using very different approaches. Unlike SWATH, iTRAQ sample preparation requires preparatory steps to covalently bind isobaric labels to peptides which usually represent 4 or 8 different experimental groups depending on the study [11–13].

Data dependent acquisition (DDA) method is often used in iTRAQ experiment, which has been widely used for relative quantitation of proteins in clinical and in vitro studies [14–17]. Usually, the iTRAQ studies have been fairly small (< 40 samples), with total protein amount > 30 µg/sample [16–19] and in general, 25 µg of total protein has been regarded as a limit for successful iTRAQ labeling. iTRAQ has not been applied for large clinical trials because of the high costs of the reagents, complexity of the study setups and propensity of iTRAQ to yield incomplete data based on stochastic selection of peptides.

SWATH is a data independent acquisition (DIA) method where the instrument deterministically fragments all precursor ions within the predefined mass-to-charge (m/z) range and acquires convoluted product ion spectra containing all the fragment ions of all the concurrently fragmented precursors [13]. This results in a data set that is continuous in both fragment ion intensity and retention time dimensions and essentially represents a digital recording of all analyzed proteins in the sample [3]. In SWATH, datasets are recorded independently and can be re-examined if the library used in downstream analyses is updated. In addition, SWATH is label free and therefore not limited to a specific number of experimental groups [13]. SWATH was first presented by Gillet et al. [13], since then multiple different articles on the subject have been published [9, 21, 22]. DIA methods are also suitable for analysis of extremely small sample amounts [9, 23], which is crucial for small and non-reproducible clinical samples, common in many clinical specialties such as ophthalmology. SWATH allows more flexible comparisons and potential savings, especially when sample amount is limited [13].

iTRAQ and SWATH both produce relative quantitation results which need further validation using other methods in order to increase the reliability of discovery proteomics. Suitable methods for this purpose include different immunochemical methods and targeted MS/MS analysis. However, ELISA assays and Western blotting are both often limited by selectivity and multiplexing possibilities with small samples [24, 25]. Targeted MS/MS analysis is currently the only mass spectrometry method which can produce absolute quantification of targeted proteins using isotopically labeled peptide standards (AQUA) or protein standard absolute quantification (PSAQ) [26, 27]. Use of isotope standards minimizes the sample preparation and mass spectrometry equipment variation thus increase the reliability and accuracy of results [28].

We compared iTRAQ and SWATH in terms of sensitivity, reliability and robustness in tear fluid samples collected from 28 patients over five time points in a clinical drug switch study. In addition, we evaluated similarity of relative quantification results to 2 stable isotope labeled protein peptides (AQUA) quantification results obtained by MicroLC-MS/MS.

## Methods

### Chemicals and materials

Acetonitrile (ACN), formic acid (FA), water (UHPLC-MS grade), triethylammonium bicarbonate buffer 1 M (TEAB), sodium dodecyl sulfate (SDS), methyl methanethiosulfate (MMTS), trifluoroacetic acid (TFA), ammonium bicarbonate (ABC) and urea were all purchased from Sigma Aldrich (St. Louis, MO, USA). Protease inhibitor cocktail (HALT) and sample cleaning tips (C18) were from Thermo Fisher Scientific (San Jose, CA, USA). BioRad DC protein quantification kit and serum albumin standard were purchased from Bio-Rad (Hercules, CA, USA) and 30 kDa filters from PALL (Port Washington, NY, USA). 8-plex iTRAQ reagent kit and TPCK treated trypsin was acquired from Sciex (SCIEX, Framingham, MA, USA).

### Sample collection and preparation

The study was approved by the Ethics Committee at Tampere University Hospital and was registered in EU clinical trials register (EudraCT Number: 2010-021039-14).

**Fig. 1:**
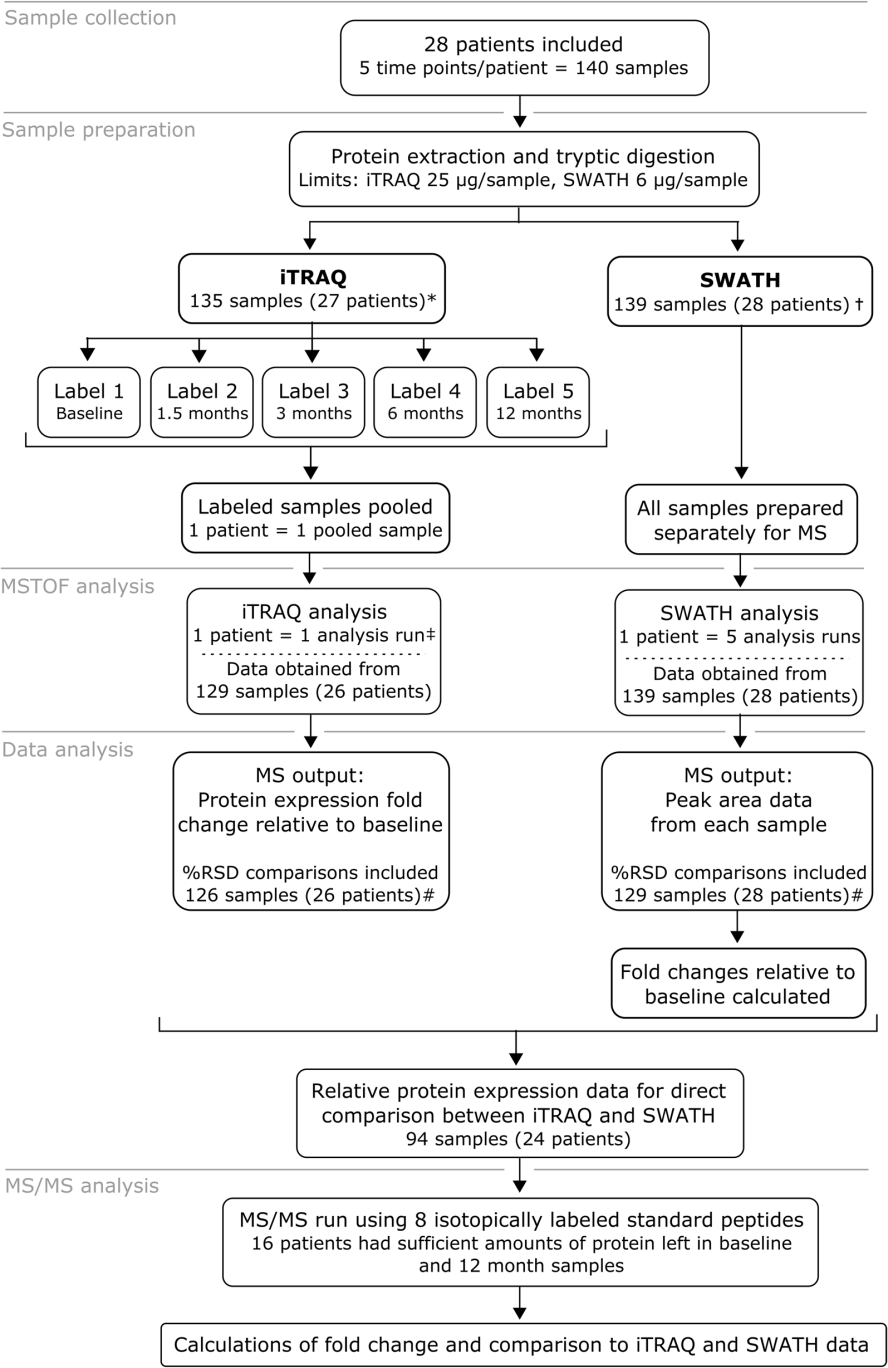
Illustration of study outline, different analyses completed in the study and sample amounts

The study outline, sample preparation and analysis are schematically presented in Fig. 1. The study was performed on 28 glaucoma patients who had been using latanoprost as topical glaucoma medication for the minimum of 2 years prior to switching to preservative-free medication. Patients’ eyes were clinically examined by an ophthalmologist and tear samples were collected with Schirmer’s strips 0, 1.5, 3, 6 and 12 months after the drug change. No topical anesthesia was used in tear sample collection and gloves were worn by all investigators handling the samples. The samples were stored at − 80 °C until processed.

### Protein extraction and enzymatic digestion

For extraction of tear proteins, Schirmer’s strips were first cut into small pieces and solubilized to 50 mM ABC solution containing HALT™ protease inhibitor, incubated on ice for 60 min and centrifuged. Total protein concentration of the supernatant was measured with Bio-Rad DC protein quantification kit, using serum albumin as a standard. For iTRAQ 25 or 50 µg and for SWATH 6–50 µg of total protein was precipitated with cold acetone (− 20 °C) overnight. The protein amounts were selected based on the manufacturer’s recommendation in iTRAQ and our previous testing of suitable injection volume of 1.5 µg total protein in 2 µl volume in SWATH. Precipitated proteins were centrifuged, liquid layer was decanted from the tubes and samples were allowed to dry for 10 min. Proteins were solubilized in 0.5 M TEAB, denatured with 0.1% SDS and reduced by Tris-(2-carboxyethyl) phosphine and set to thermo mixer for 60 min of incubation at + 60 °C. After reduction samples were transferred into Pall Nanosep^®^ 30 kDa MWCO centrifugal devices with low protein binding membrane and flushed three times with 200 µl of 75% urea solution for additional denaturation. Cysteine residues were blocked by MMTS at room temperature in dark. Samples were flushed with additional urea solution twice, and urea was removed with 50 mM ABC. Samples were then subjected to enzymatic digestion (trypsin:protein ratio 1:25 µg) for 16 h at + 37 °C. The following day samples were flushed with 50 Mm ABC to remove peptides from the filter and dried in speed vacuum. SWATH samples were solubilized to 0.1% TFA and desalted with C18 filter tips. Desalting was done by first cleaning the filter by aspirating 50% ACN and 0.1% TFA through the tip, then sample was aspirated 7 times, followed by cleaning aspiration with 5% ACN. Final elution was done by 80% ACN and 0.1% FA. Samples were dried in speed vacuum and stored at − 20 °C until used for iTRAQ labeling or reconstituted to loading solution (5% ACN, 0.1% FA) before NanoRPLC-MSTOF SWATH analysis. SWATH analysis samples were diluted to equal concentrations before injection to the instrument.

### iTRAQ labeling

Each iTRAQ reagent (113, 114, 115, 116, 117, 118, 119, 122 isobaric tags) was reconstituted in ethanol, and peptide samples were reconstituted in iTRAQ dissolution buffer. Samples were labelled with the iTRAQ reagents one patient at a time (5 samples = 5 labels per patient). The labelled samples were incubated for 2 h at room temperature with interval mixing (alternating 1 min static and 15 min at 1200 rpm), centrifuged at 13,500 rpm for 5 min, and pooled (Fig. 1). The pooled samples were dried by vacuum centrifugation. Sample clean-up and desalting was carried out using Macro Spin Column Filters (Nest Group Inc, Southborough, MA) and washing samples several times with ACN and TEAB buffers. The samples were dried in vacuum centrifugation and stored at − 20 °C until analyzed.

### NanoRPLC-MSTOF

NanoLC-MSTOF parameters used in iTRAQ analysis have been published before in [17] and SWATH method has been published before [9]. Digested peptides were analyzed by Nano-RPLC-MSTOF instrumentation using Eksigent 425 NanoLC coupled to high speed TripleTOF™ 5600 + mass spectrometer (Ab Sciex, Concord, Canada). A capillary RP-LC column (cHiPLC^®^ ChromXP C18-CL, 3 µm particle size, 120 Å, 75 µm i.d × 15 cm, Eksigent Concord, Canada) was used for LC separation of peptides. Samples were first loaded into trap column (cHiPLC^®^ ChromXP C18-CL, 3 µm particle size, 120 Å, 75 µm i.d × 5 mm) from autosampler and flushed for 10 min at 2 µl/min (2% ACN, 0.1% FA). The flush system was then switched to line with analytical column. Tear samples were analyzed with 120 min 6 step gradient using eluent A: 0.1% FA in 1% ACN and eluent B: 0.1% FA in ACN (eluent B from 5 to 7% over 2 min, 7 to 24% over 55 min, 24 to 40% over 29 min, 40 to 60% over 6 min, 60 to 90% over 2 min and kept at 90% for 15 min, 90 to 5% over 0.1 min and kept at 5% for 13 min) at 300 nl/min.

Key parameters for TripleTOF mass spectrometer in iTRAQ and SWATH ID library analysis were: ion spray voltage floating (ISVF) 2300 V, curtain gas (CUR) 30, interface heater temperature (IHT) + 125 °C, ion source gas 1 13, declustering potential (DP) 100 V. Library for SWATH analysis was created from the same samples by information dependent-acquisition (IDA) method and relative quantitation analysis was done by SWATH method. All methods were run by Analyst TF 1.5 software (Ab Sciex, USA). For DDA parameters, 0.25 s MS survey scan in the mass range 350–1250 m/z were followed by 60 MS/MS scans in the mass range of 100–1500 Da (total cycle time 3.302 s). Switching criteria were set to ions greater than mass to charge ratio (m/z) 350 and smaller than 1250 (m/z) with charge state 2–5 and an abundance threshold of more than 120 counts. Former target ions were excluded for 12 s. IDA rolling collision energy (CE) parameters script was used for automatically controlling CE. SWATH quantification analysis parameters were the same as for SWATH ID, with the following exceptions: cycle time 3.332 s and MS parameters set to acquire an MS scan in the range of 350–1250 Da followed by 40 MS/MS scans of 15 Da windows spanning the mass range 350–1250 Da.

### MicroLC-MS/MS analysis with AQUA peptides

Sixteen patients were selected for MS/MS analysis. Selection of patients was based on what samples had sufficient amount tear fluid left for MS/MS analysis after iTRAQ and SWATH analyses. We selected 8 proteins and one peptide from each for MicroLC-MS/MS analysis (Table 1). Sample preparation was similar to SWATH samples and AQUA peptides were added in the final elution step before injecting to MS/MS analysis. Concentration of AQUA peptide was matched to the mean concentrations of each peptide in the tear samples. Corresponding isotopically labeled peptides were purchased from Sigma-Aldrich (purity > 95%).

**Table 1:** Proteins and their specific peptides and fragments used in MS/MS quantification analysis

Protein	Peptide sequence	Mass (Da)	Corresponding isotope
LYZ	STDYGIFQINSR	1400.7	(H)STDYGIFQINS(R_C13N15_)(OH)
S100A6	LQDAEIAR	915.5	(H)LQDAEIA(R_C13N15_)(OH)
PROL1	LNSPLSLPFVPGR	1396.8	(H)LNSPLSLPFVPG(R_C13N15_)(OH)
TF	SASDLTWDNLK	1249.5	(H)SASDLTWDNL(K_C13N15_)(OH)
YWHAZ	GIVDQSQQAYQEAFEISKK	2169.1	(H)GIVDQSQQAYQEAFEISK(K_C13N15_)(OH)
S100A9	LGHPDTLNQGEFK	1455.7	(H)LGHPDTLNQGEF(K_C13N15_)(OH)
CST4	IIPGGIYDADLNDEWVQR	2074.0	(H)IIPGGIYDADLNDEWVQ(R_C13N15_)(OH)
YWHAE	EAAENSLVAYK	1194.6	(H)EAAENSLVAY(K_C13N15_)(OH)

Tear samples were analyzed with Sciex 6500 + MSTrap which was coupled to NanoLC 425 with 1–10 µl/min microLC flow cell. Acclaim™ PepMap™ C18, analytical C18, 300 Å, 5 µm, 0.3 × 150 mm HPLC column was purchased from Thermo Fisher scientific. MicroLC utilized a 42 min 6 step gradient using eluent A: 0.1% FA in MQ and eluent B: 0.1% FA in ACN (eluent B from 10 to 30% over 22 min, 30 to 50% over 8 min, 50 to 80% over 2 min, kept at 80% for 5 min, 80 to 10% over 0.2 min and kept at 10% for 5 min, at 5 µl/min. MSTrap settings were as follows; Curtain gas: 30, Spray voltage: 5300, Collision gas: medium, Temperature: 150 °C, Ion source gas 1: 20, Ion sourece gas 2: 20, were set the same for all peptides. Collision energy was set to 30 for all fragments and corresponding isotopes.

### Data analysis

ProteinPilot software version 4.0.8085 (AB Sciex, Canada) was used for database searching (IPI v. 3.87) for building library for SWATH analysis and iTRAQ analysis. Some important settings in the Paragon search algorithm in ProteinPilot were configured as follows. Sample type: identification, Cys-alkylation: MMTS, Digestion: Trypsin, Instrument: TripleTOF 5600 +, Search effort: thorough ID. False discovery rate (FDR) analysis was performed in the ProteinPilot and FDR < 1% was set for protein identification. Peptide identification limit was set to 99%. The data from all the identification runs were combined as a batch and used for library creation.

PeakView^®^ software 2.0 with SWATH was used to assign the correct peaks to correct peptides in the library. iRT peptides (Biognosys, Switzerland) was used for retention time calibration with PeakView. 1–15 peptides per protein were selected to be used in SWATH quantification. All shared peptides were excluded from analysis. SWATH plug-in FDR analysis was used to select the proper peptides for use in quantification. For all comparison data between iTRAQ and SWATH all proteins with relative quantitation data based on single peptide were removed from results.

All statistical analyses were performed with Excel or R statistical software version 3.2.3 (R Core Team. Foundation for Statistical Computing, Vienna, Austria. URL: https://www.R-project.org/). Relative standard deviations (% RSD) were calculated from two replicate MS analyses in order to assess repeatability of iTRAQ and SWATH. Duplicate proteins with identical gene names were merged, keeping only the protein name with the highest ranking (number of proteins after duplicate check, iTRAQ n = 667, SWATH n = 680, Additional file 1).

For MS/MS quantification method we used 3 point smoothing in peak integration and all 3 peptide fragment chromatograms were combined for each protein. Quantified peptides areas were normalized against their corresponding isotopically labeled standard peptide peak areas to remove instrument variability. Analysis method was validated in terms of sample preparation, linearity and repeatability.

## Results

All of the iTRAQ and SWATH analyses were performed using identical TripleTOF 5600 + MS and NanoLC settings. A total of 14 days of instrument time was required for iTRAQ analyses and 35 days for SWATH analyses. Altogether 140 tear samples were processed, however, iTRAQ analyses were performed with 135 samples, as protein concentration of the remaining 5 samples was below the manufacturer’s suggested limit of 25 µg/sample. The corresponding limit for SWATH analysis was 6 µg of protein and only one sample fell below this limit. We obtained corresponding data for 94 tear samples iTRAQ and SWATH analyses (Fig. 1).

**Fig. 2:**
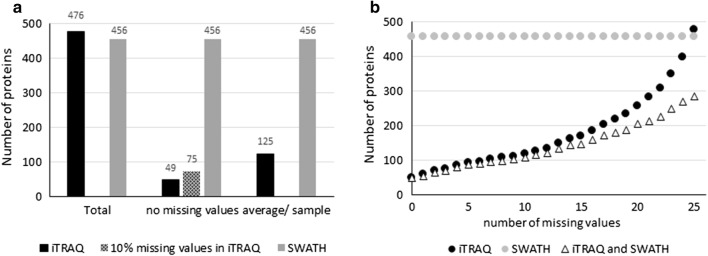
Protein quantification summary. **a** Number of analyzed proteins with iTRAQ and SWATH methods. **b** The effects of allowing missing values on the number of quantified proteins using iTRAQ (cumulative)

We identified a total of 667 different proteins using iTRAQ in total of 13,220 peptides from 135 samples, corresponding to 310,452 identified spectra, and 477 proteins had more than two peptides and were quantified. With SWATH we identified a total of 11,791 peptides from 60 samples, corresponding to 285,263 identified spectra in an assembly of 680 proteins using False discovery rate of 1.0%. From this library, 456 proteins had more than two distinct peptides in all analyzed tear samples. The average amount of proteins identified in all time points of one patient by iTRAQ was 125, (range 59–258) and 49 proteins were quantified in all samples. Moreover, 283 proteins were quantified with both analysis methods. iTRAQ is known to generate data with missing values, and we observed how, by allowing missing values, the number of quantified proteins increased (Fig. 2b). The SWATH protein library was more representative of the study as a whole. iTRAQ and SWATH libraries with peptide information are presented in Additional file 1.

### Data quality assessment

**Fig. 3:**
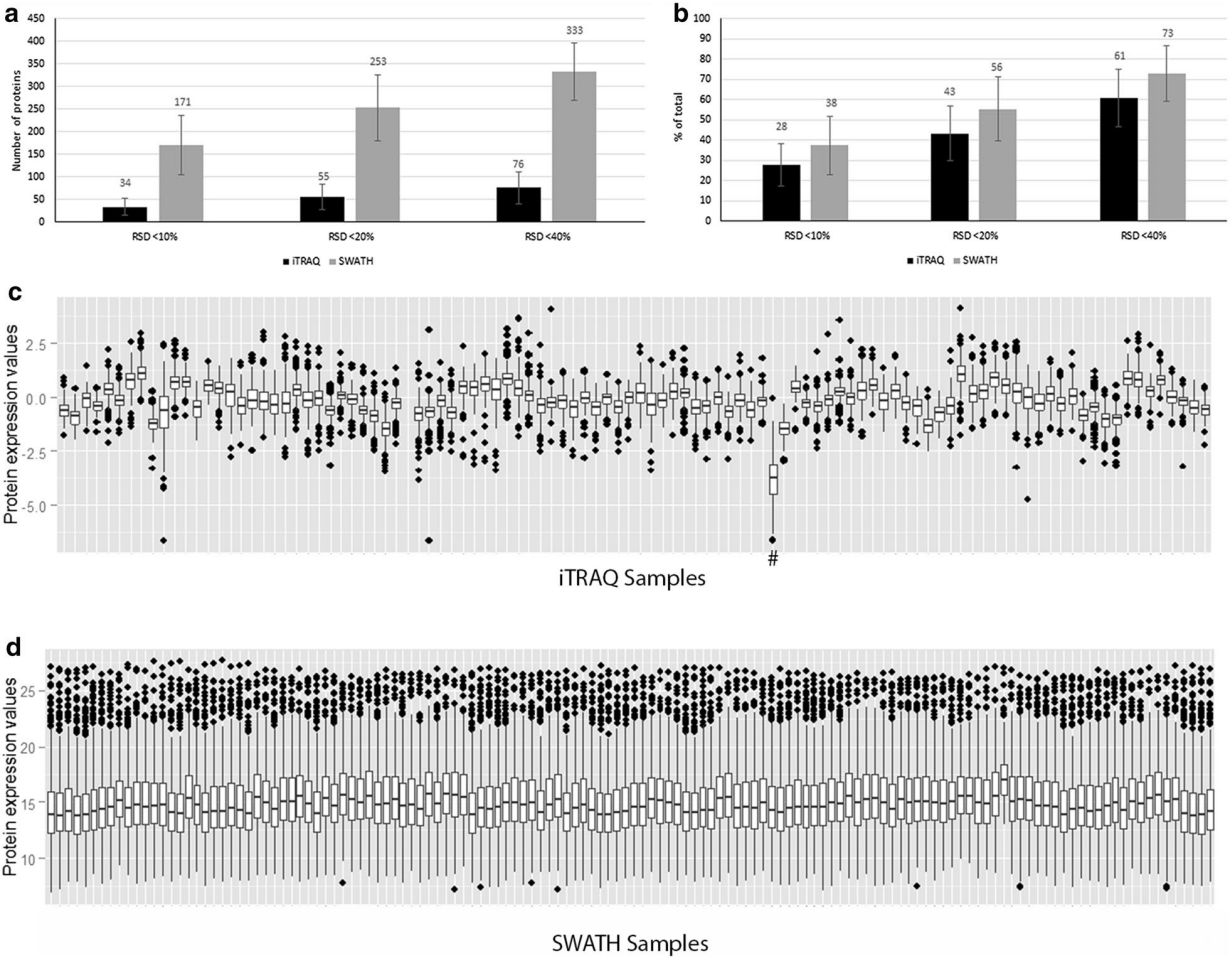
Data quality assessment. **a** Total number of relatively quantified proteins and **b** proportion of total analyzed proteins in technical replicates with RSD < 10, < 20 and < 40% (Mean ± SD, n(iTRAQ) = 125, n(SWATH) = 456). Distribution of proteomic results of individual samples analyzed with **c** iTRAQ, # one sample deviated from the rest in iTRAQ and **d** SWATH, expressed as mean of technical replicates (log_2_-scale)

Each tear sample was run twice. In these replicate MS analyses, iTRAQ quantified an average of 125 and SWATH 456 proteins. In order to assess repeatability, we calculated and compared % RSD. At different % RSD cut-off levels SWATH consistently showed higher number of proteins in relation to total number of quantified proteins (Fig. 3a, b).

To evaluate the effect of sample preparation and instrument stability, we visualized protein expression variation of individual samples by boxplots (Fig. 3c, d). Variability between sample distributions was minor and medians were consistently close to zero, however, distribution variation in iTRAQ was larger than in SWATH.

**Fig. 4:**
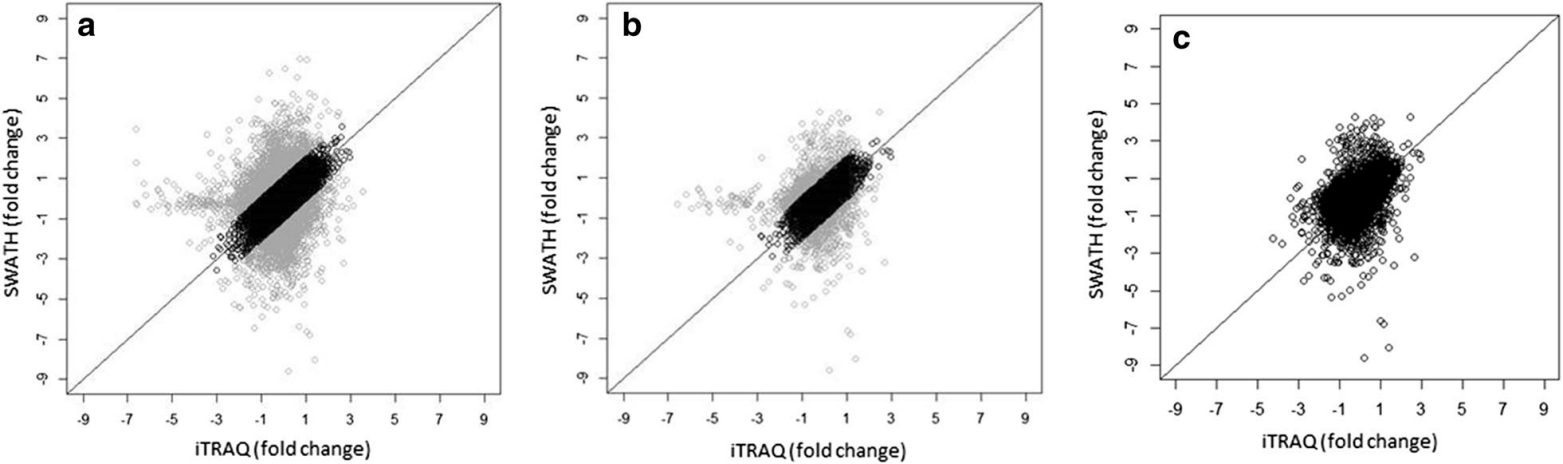
Variation of the results in iTRAQ and SWATH. Y-and x-axes represent the expression value (log_2_-scaling) in each sample. **a** All results quantified in 94 samples by both methods, n = 14,473 relative fold change values (Spearman’s rank correlation coefficient r^2^ = 0.345). **b** 49 proteins quantified in all iTRAQ samples correlated against SWATH results, n = 4606 (r^2^ = 0.414). **c** 49 proteins with one outlier sample removed (r^2^ = 0.420).** a** and** b** highlighted parts represent the < 1 fold change difference between results

To correlate the fold change values between the two methods we compared the protein expression values obtained by iTRAQ to those of SWATH. There were altogether 103 samples and 14 527relative changes in protein levels where this comparison was possible (Fig. 4). The relative changes were similar between two methods: 70.6% of all proteins (and 76.1% of the 49 proteins seen in all samples) had less than onefold difference between corresponding iTRAQ and SWATH results (Fig. 4a, b). One iTRAQ sample was clearly deviating from the rest (Fig. 4a, b) and its removal from the data increased the correlation of iTRAQ and SWATH (Fig. 4c). This deviation was also seen in the median and range in comparison to the other samples in iTRAQ box plot (Fig. 3c).

### MS/MS validation using isotopically labeled peptides

Finally, we validated the SWATH and iTRAQ results using MicroLC-MS/MS analysis with AQUA peptides. We selected 8 proteins with 1 peptide per protein and two different time-points (first and last) from 16 patients for the targeted quantification with isotopically labeled peptides. MS/MS method sample preparation together with instrument variation % RSD was < 15% for all peptides and instrument repeatability alone < 5%. Linear range was confirmed in the range of 50 fold change and all peptides showed linearity of r^2^ > 0.99. We calculated the protein expression fold change between first and last sample collection time points and compared it to SWATH-MS and iTRAQ results respectively. MS/MS fold change comparison was possible with 114 values in SWATH analysis and 110 values with iTRAQ. This comparison showed similar results between the two methods with good correlation to MS/MS. Median difference to MS/MS values were 0.33 [0.60 ± 0.79 (Mean ± SD)] using iTRAQ and 0.45 [0.62 ± 0.74 (Mean ± SD)] with SWATH-MS. Illustration of all 8 proteins results are represented in Additional file 2.

### Clinical correlation of LYZ and S100A6 expression changes in two methods

**Fig. 5:**
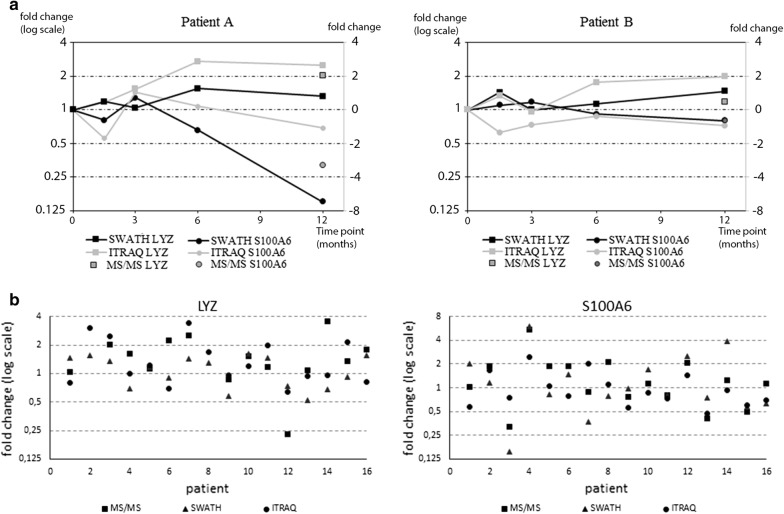
Proteomic fold changes over time and comparison to targeted MS/MS analysis of specific peptides of two selected proteins (LYZ and S100A6). **a** Changes in LYZ and S100A6 protein expression levels over time relative to baseline expression, analyzed using iTRAQ, SWATH and MS/MS in two representative patients. **b** Fold change results between baseline sample and 12 month sample in 16 patients. Presented proteins were selected based on their known biological function. iTRAQ and SWATH results are based on average of two replicate injections and AQUA peptides analysis on single technical replicate analysis

In order to verify biological validity of the results we selected two known tear fluid biomarkers for longitudinal analysis. Lysozyme (LYZ) and S100A6 protein expressions have been linked to dry eye disease and glaucoma drug adverse effects; LYZ expression decreases while S100A6 increases in response to these conditions [6, 7, 20]. Both proteins were quantified in all samples using both analysis methods. In addition, our results demonstrate good correlation between SWATH and iTRAQ (Fig. 5a). Based on protein expression levels of LYZ and S100A6 we were able to demonstrate that both patients are showing improvement on the ocular surface and that patient A reacts faster to the drug switch than patient B. Figure 5b result highlights the patient specific response, as we can see that in most cases LYZ is increased while S100A6 expression has gone down. Furthermore, patients appear to have very specific responses and the level of response varies between patients.

## Discussion

This study compared two commonly used methods for relative quantitative proteomics (iTRAQ and SWATH) to demonstrate the particular value of SWATH analysis when confronted with the issues often found in clinical studies. In addition, we compared the results to isotopically labeled peptide standards (AQUA) using MicroLC-MS/MS which showed that accuracy of SWATH and iTRAQ are at similar level and correlated well with absolute concentration measured with isotopically labeled peptide standards. For studies conducted in controlled conditions with few time points, iTRAQ is useful in detecting relative changes in small samples such as tear fluid [6, 7, 28]. However, increasing numbers of patients and follow-up time points in clinical studies mean that label-free technologies such as SWATH provide a more consistent number of quantified results across all samples and thus is more suitable for the discovery of putative biomarkers.

Currently, the highest number of proteins identified from tear fluid samples is 1543. This result was obtained by using pooled samples and LC fractionation [6]. However, in clinical studies such as ours, study design requires individual sample preparation and analysis. Despite these restrictions, by combining iTRAQ and SWATH data we were able to identify 1055 and quantify 959 proteins (620 with two peptides). Using iTRAQ only, we quantified a total of 667 (477 using two peptides) proteins without pooled samples and LC fractionation, which is almost double of what has been previously reported for tear fluid [20]. In addition, the average (125) and maximum (258) number of proteins quantified per patient was somewhat higher than reported previously [20, 29]. Proteome analysis using SWATH has been able to relatively quantify 785 proteins from a 1 µl tear sample [9]. This technique allowed us to quantify 456 (680 including one peptide results) proteins in all of the tear fluid samples studied. Of these 477 (iTRAQ) and 456 proteins (SWATH), 283 were shared, leaving 173 proteins quantified only by iTRAQ and 173 only by SWATH. Similar difference between iTRAQ and SWATH data has been demonstrated previously in other types of samples [30, 31].

In our study the maximal number of proteins quantified with iTRAQ in all samples of one patient was 258. However, only 49 identical proteins were quantified in all samples of the 26 patients. This feature of iTRAQ has been characterized previously also by [20, 29]. The incomplete data in this type of study is generated based on stochastic selection of peptides [11, 31]. Various normalization and missing value imputation methods can correct variation and incomplete data to a certain extent [32, 33]. However, imputation methods in general have not gained much popularity because their validation is challenging [34]. Therefore, we chose not to use imputation algorithms to complete the data in this study.

When correlating the data obtained by using iTRAQ and SWATH we found that they were in a fairly good agreement. 70.6% of the 283 proteins quantified by both methods showed less than onefold change difference to each other and had a correlation coefficient of r^2^ = 0.414. Several comparisons of iTRAQ and SWATH have been reported [30, 31, 35], but their results cannot be directly compared with ours due to the differences in study design and instrumentation. Our correlation coefficient was between previous study results [31, 35], showing correlation of r^2^ = 0.726 and r^2^ = 0.312. Data variation seen in boxplots is probably due to sample processing and instrument variation in the SWATH analysis and labeling and instrument variation in iTRAQ analyses.

The differences in fold change values between iTRAQ and SWATH suggest that there is variation in the accuracy of the results. Overall, SWATH analysis yielded a wider range of fold change values than iTRAQ. Fold change underestimation in iTRAQ has been demonstrated previously [31, 36, 37] and is due to the mixed MS/MS in complex samples where two precursor ions are selected to the same window for MS/MS. As a result, the average of two reporter ion intensities, instead of one, determines a single peptide fold change.

MRM (multiple reaction monitoring) using AQUA peptides is considered to be the gold standard and only method for absolute quantification of proteins using mass spectrometry [28]. Results showed that iTRAQ and SWATH median and mean values were close to each other but standard deviation was higher in iTRAQ. When focusing on single protein results, some differences between the methods were observed but it was not possible to identify a common nominator for the differences. Based on AQUA peptide results we can conclude that using these peptides, both methods are equally accurate and are able to produce reliable relative quantification data, although SWATH can gain higher number of quantified results per sample with same reliability. Further validation of the results using western blotting or ELISA assays were not possible due to the low sample amounts.

As a clinical example, we selected two known markers of ocular surface pathologies [6, 19] proteins (LYZ and S100A6) for closer examination and compared the protein expression change over time using iTRAQ and SWATH. After the drug switch, we found an increase in LYZ expression together with a decrease in S100A6 expression as expected if drug switch is beneficial for the patient. SWATH and iTRAQ both revealed similar changes and the results correlated well to MS/MS data. However, as also noted previously, iTRAQ often underestimated fold changes [31, 36].

## Conclusions

Label-free techniques are becoming accepted as suitable tools for clinical proteomic analyses, and it has been suggested that future workflow should use DDA methods only in the preliminary hypothesis stage [38, 39]. Our data supports this view by demonstrating benefits of SWATH in a study having typical clinical study design. Although SWATH has several advantages over iTRAQ, there are still some remaining issues. SWATH analysis is time consuming and will take at least twice as much instrument time as iTRAQ analysis. SWATH requires a spectrum library to be created before analyzing any specific type of samples, which consumes instrument time and may require additional samples. Despite the additional work at the initial stages, once the library is created, it can be used afterwards for similar samples. Overall the complexity of data is far greater using SWATH since it is not limited to specific study design, which allows basically unlimited number of hypotheses to be tested against the data. This highlights the importance of bioinformatics as comparisons between time points, patients and patient data leads to enormous data amounts and can easily lead to false positives. On the other side wider range of comparisons means that one clinical study can provide much more information. In addition to higher data completeness and smaller % RSD variation, SWATH is showing reliable expression level changes between time points. Overall, for larger and complex clinical studies our research results support the use of label free methods.

## Additional files

**Additional File 1.** List of all analyzed proteins and the number of peptides used for relative quantitation of each protein.

**Additional File 2.** Fold changes values for 8 proteins which were measured with all three different analysis methods.

## Abbreviations

SWATH-MS: sequential window acquisition of all theoretical fragment ion spectra mass spectrometry
iTRAQ: isobaric tags for relative and absolute quantitation
DDA: data dependent analysis
DIA: data independent analysis
LC-MS/MS: liquid chromatography tandem mass spectrometry
