# Supplementary figures and images for: Comparison of iTRAQ and SWATH in a clinical study with multiple time points
Source: Clin Proteomics. 2018 Jul 30;15:24. doi: 10.1186/s12014-018-9201-5 (PMC6065059; doi:10.1186/s12014-018-9201-5)

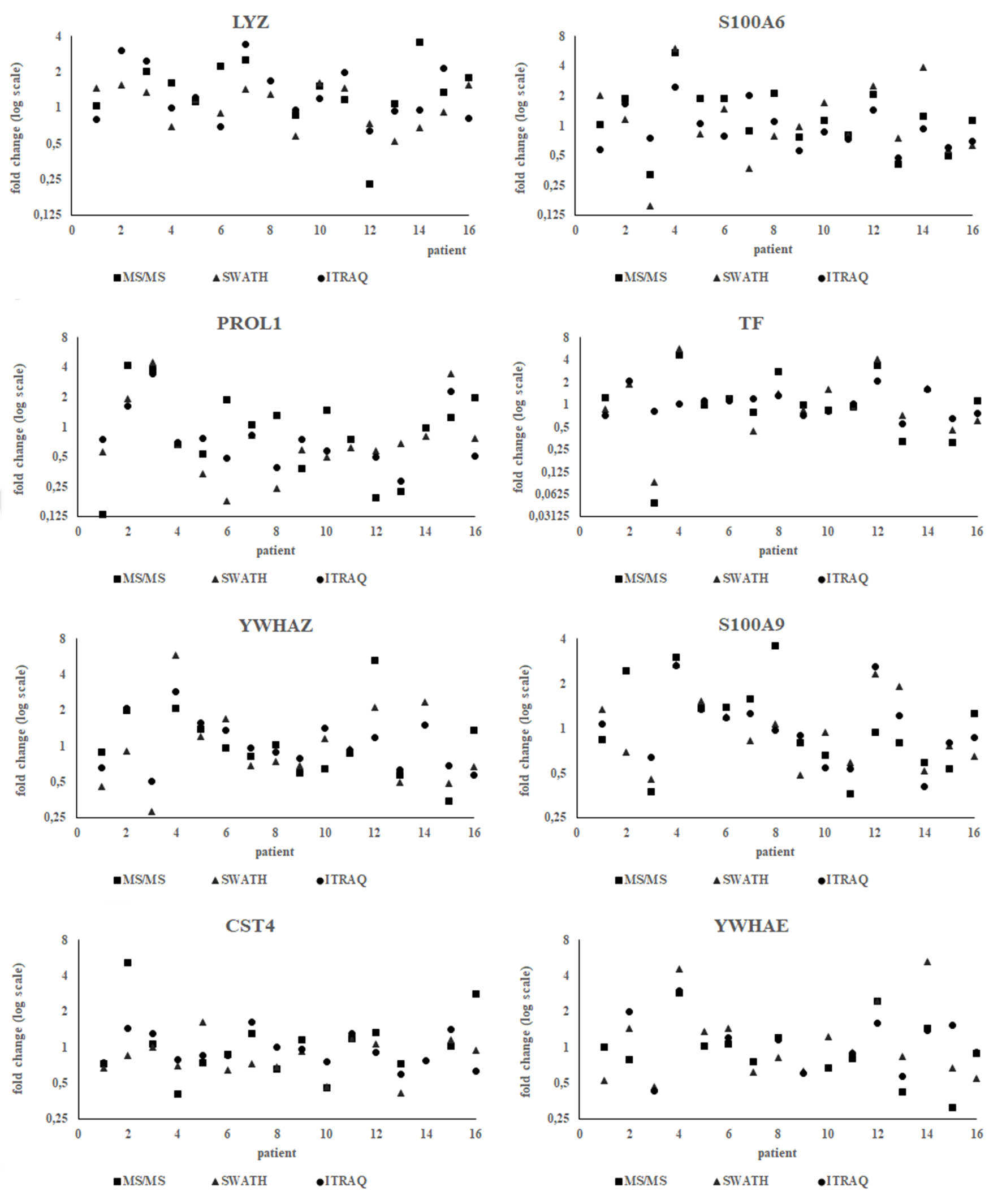

Supplement: Supplementary file 2 — Additional File 2. Fold changes values for 8 proteins which were measured with all three different analysis methods. [file 12014_2018_9201_MOESM2_ESM.tif]
